# Job Stress Does Not Predict Safety Outcomes But Safety Culture Does: Insights From Emergency Nurses in Iran

**DOI:** 10.1155/jonm/6410188

**Published:** 2026-08-26

**Authors:** Alam Abbasi Yazdi, Javad Moghri, Jamshid Jamali, Elahe Hooshmand

**Affiliations:** ^1^ Student Research Committee, Mashhad University of Medical Sciences, Mashhad, Iran, mums.ac.ir; ^2^ Social Determinants of Health Research Center, Mashhad University of Medical Sciences, Mashhad, Iran, mums.ac.ir; ^3^ Department of Health Management and Economic Sciences, School of Health, Mashhad University of Medical Sciences, Mashhad, Iran, mums.ac.ir

**Keywords:** emergency nursing, Iran, nursing stress, patient safety, PLS-SEM, safety culture

## Abstract

**Background:**

Emergency departments are high‐stress environments where nursing performance directly impacts patient safety. While job‐related stress is assumed to influence safety outcomes, the mediating role of patient safety culture (PSC) remains unclear, especially in resource‐limited healthcare systems.

**Methods:**

This cross‐sectional study surveyed 304 emergency nurses in two teaching hospitals in Iran using validated Persian versions of the Nursing Stress Scale and the Hospital Survey on Patient Safety Culture. Relationships among job‐related stress, PSC, and perceived patient safety outcomes (PSOs) were analyzed using partial least squares structural equation modeling (PLS‐SEM).

**Results:**

Job‐related stress was not significantly associated with PSC (*β* = −0.339, *p* = 0.125) or PSO (*r* = −0.043, *p* > 0.05), and no mediation effect was observed. However, PSC demonstrated a strong and significant positive correlation with PSO (*r* = 0.807, *p* < 0.001), highlighting its potential as a target for safety improvement.

**Conclusions:**

Although the hypothesized mediation model was not supported, the findings underscore the central role of safety culture in promoting better patient outcomes. Reporting null associations contributes to a more balanced understanding of stress–safety dynamics, particularly in middle‐income healthcare settings. Future studies should consider organizational context and objective safety indicators.

## 1. Introduction

Patient safety is a fundamental component of healthcare quality and has become a global concern across all clinical settings [1]. Emergency departments (EDs), in particular, pose elevated risks for medical errors due to their fast‐paced, unpredictable, and high‐pressure environments. Nurses working in EDs are often the first point of contact for patients and are continuously required to make rapid decisions under stressful conditions [2]. These circumstances increase their vulnerability to job‐related stress and fatigue, both of which can adversely impact the quality and safety of patient care [3]. According to the World Health Organization, unsafe medical practices lead to millions of preventable injuries and deaths annually, and a significant portion of these events are related to human and systemic factors within nursing care [4].

Job‐related stress is a well‐documented occupational hazard among nurses [5]. Numerous studies across Europe, North America, and Asia have reported associations between high levels of stress and undesirable patient outcomes, including increased medication errors, poor communication, and low‐quality documentation [3, 6–8]. In parallel, healthcare organizations have increasingly focused on the concept of patient safety culture (PSC)—an organizational framework encompassing shared values, attitudes, and behaviors that support safe practices [9]. A positive PSC encourages error reporting, teamwork, nonpunitive responses to mistakes, and managerial support for safety improvement [10, 11].

Several international studies have demonstrated the independent effects of both job‐related stress and PSC on patient outcomes. For example, research in Taiwan and South Korea has shown that high PSC levels are associated with reduced adverse events and greater reporting transparency [12–14]. However, there is growing interest in understanding how PSC may operate as a mediating factor, potentially mitigating the impact of stress on performance and safety. Despite this theoretical possibility, empirical evidence on this interaction remains limited and somewhat inconsistent across contexts [15]. This hypothesized mediating role of PSC is theoretically supported by organizational behavior models, which suggest that a strong safety culture can buffer the negative effects of occupational stress by promoting shared values, open communication, and team‐based coping mechanisms [16]. Some studies support the buffering role of PSC, while others report no significant mediation effect, especially in resource‐constrained or underfunded settings [16–18].

Moreover, many of these studies have been conducted in high‐income countries with well‐established safety protocols, which may not reflect the realities of middle‐ or low‐income healthcare systems. In such settings, chronic understaffing, low error‐reporting rates, and limited support structures can exacerbate stress and undermine safety efforts [19]. There is a lack of comprehensive research that explores these dynamics in countries with constrained healthcare resources.

Given the mixed evidence in the literature, this study adopts an exploratory approach to examine the interrelationships between job‐related stress, PSC, and patient safety outcomes (PSOs) among emergency nurses in Iran. Rather than assuming a fixed causal path, the study explores whether PSC may serve as a mediating factor between stress and outcomes, recognizing the complexity and contextual nature of these relationships.

Although this study was conducted in Iran, its findings contribute to a broader understanding of how stress and organizational culture interact to shape PSOs in emergency settings globally. Moreover, understanding when and why expected relationships do not emerge is equally important for developing realistic and context‐sensitive patient safety interventions. By examining these dynamics in a real‐world, high‐demand healthcare environment, the study offers insights relevant to frontline nursing care across diverse cultural and economic systems.

In addition, this study contributes to the literature by explicitly testing a mediation model using partial least squares structural equation modeling (PLS‐SEM) and by reporting nonsignificant findings, which are often underreported but are essential for refining theoretical assumptions and reducing publication bias.

## 2. Methods

### 2.1. Study Design and Setting

This cross‐sectional, analytical study was conducted in 2024 in two major teaching hospitals in Mashhad, Iran. Mashhad is the second most populous city in the country and serves as a regional referral hub for medical care in northeastern Iran. The city has five large teaching hospitals affiliated with Mashhad University of Medical Sciences. Among them, Imam Reza and Ghaem hospitals—two of the busiest and most comprehensive emergency care centers—were randomly selected for inclusion in the study. These hospitals receive patients from across the city and neighboring provinces, making them representative of large, high‐volume EDs in public Iranian healthcare settings.

### 2.2. Population and Sample Size

The target population included all registered nurses working in the EDs of the selected hospitals. At the time of data collection, approximately 350 nurses were eligible. Using Cochran’s formula for finite populations with a 95% confidence level and 5% margin of error, the required minimum sample size was 184. To enhance robustness and account for potential nonresponse, 340 nurses were invited to participate, and 304 complete and valid responses were collected.

### 2.3. Sampling Method

A cluster random sampling technique was used. First, two hospitals were randomly selected out of five eligible teaching hospitals. Within each hospital, all emergency nurses meeting the inclusion criteria were invited to participate through nursing supervisors and internal networks. Inclusion criteria were as follows: (1) at least 6 months of continuous work experience in the ED; (2) direct involvement in patient care; and (3) willingness to complete the questionnaire anonymously. Participation was voluntary and based on informed consent.

In this study, job‐related stress was defined conceptually as the extent to which nurses perceive their work conditions as psychologically taxing, and operationally measured using the Nursing Stress Scale (NSS). PSC referred to the shared values, norms, and practices within hospital units that promote safety, assessed via the Hospital Survey on Patient Safety Culture (HSOPSC). PSOs were operationalized as perceived safety outcomes using two subscales from the HSOPSC—frequency of event reporting and overall perception of safety—rather than objective safety indicators.

Cluster random sampling was used to ensure representativeness across two distinct teaching hospitals with different patient volumes, staff workloads, and administrative structures, thereby increasing the external validity of the findings.

### 2.4. Data Collection

Data collection was conducted using self‐administered paper‐based questionnaires. Participants were invited through nursing supervisors and completed the questionnaires voluntarily during nonbusy working hours within their departments. Participation was anonymous, and no financial or material compensation was provided.

### 2.5. Instruments

Two validated questionnaires were used:

1. NSS: This 34‐item instrument measures job‐related stress in nurses across seven subscales: death and dying, conflict with physicians, inadequate preparation, lack of support, peer conflict, workload, and treatment uncertainty. Each item is scored on a 4‐point Likert scale (0 = *never* to 3 = *very often*). The Persian version has been previously validated with a Cronbach’s alpha of 0.92 (Ghahremani and Ghorbani, 2011).
2. HSOPSC: Developed by the Agency for Healthcare Research and Quality (AHRQ), this tool contains 42 items measuring 10 dimensions of PSC and 2 dimensions often interpreted as PSOs: (a) frequency of event reporting and (b) overall perceptions of safety. Responses are rated on 5‐point Likert scales. The Farsi version was validated in prior studies in Iranian hospitals (Javad et al., 2012).

Note on outcome variable:

As no direct objective outcome data (e.g., incident reports) were accessible, PSOs were operationalized as perceived safety outcomes using two subscales from the HSOPSC—frequency of event reporting and overall perception of safety. This approach has been adopted in several previous studies where objective safety outcomes were not accessible, and perceived safety indicators were used as proxies (14‐13). However, it should be noted that deriving both PSC and PSO variables from the same instrument may introduce common method bias. The normality of variables was assessed using the Kolmogorov–Smirnov test. Normality was tested for total scores and all subscales of the NSS and HSOPSC instruments. The results indicated that none of the distributions met normality assumptions (*p* < 0.05), justifying the use of nonparametric tests and PLS‐SEM.

### 2.6. Data Analysis

Data were analyzed using SPSS Version 25 and Smart PLS Version 3. Descriptive statistics (mean, standard deviation, and frequencies) were calculated. The normality of variables was assessed using the Kolmogorov–Smirnov test. Due to nonnormal distribution, Spearman correlation was used to examine the relationships between job‐related stress and PSC.

To evaluate the hypothesized mediation effect of PSC in the relationship between job‐related stress and PSOs, PLS‐SEM was applied. PLS‐SEM is a robust technique that enables modeling complex relationships among latent constructs and is suitable for small to medium sample sizes without requiring normality assumptions. It also permits the estimation of both direct and indirect (mediated) effects. It should be noted that correlation coefficients and path coefficients are derived from different analytical approaches. While Spearman correlation assesses direct bivariate relationships, PLS‐SEM estimates relationships between latent constructs within a structural model, which may result in differences in magnitude and statistical significance.

While PLS‐SEM does not offer global fit indices like covariance‐based SEM, it was deemed appropriate for this study due to the small to medium sample size, nonnormal data distribution, and the exploratory nature of the model. This methodological choice is acknowledged as a limitation and future studies may consider confirmatory approaches for mediation testing.

### 2.7. Ethical Considerations

The study was approved by the Research Ethics Committee of Mashhad University of Medical Sciences (IR.MUMS.REC.1401.409). All participants provided informed consent, and their anonymity and confidentiality were preserved throughout the study.

## 3. Results

### 3.1. Participant Characteristics

A total of 304 emergency nurses participated in the study, yielding a response rate of approximately 89%. Table 1 presents the demographic and professional characteristics of the participants. The majority were female (76.6%), married (63.5%), and held a Bachelor’s degree (94.1%). The mean age was 31.13 ± 6.42 years. Most nurses (84.2%) had fewer than 10 years of total clinical experience, and the average duration of work in the ED was 3.13 ± 2.41 years. Participants worked an average of 47.89 ± 12.84 h per week. Additional work‐related characteristics of the participants, including age, weekly working hours, and clinical experience, are presented in Table 2.

**TABLE 1:** Demographic characteristics of the participants (*n* = 304).

Characteristic	*n* (%)
Gender: Male	71 (23.4%)
Gender: Female	233 (76.6%)
Age 20–30	151 (49.7%)
Age 31–40	122 (40.1%)
Age > 41	31 (10.2%)
Marital Status: Single	111 (36.5%)
Marital Status: Married	193 (63.5%)
Education: Bachelor’s	286 (94.1%)
Education: Master’s or PhD	18 (5.9%)

**TABLE 2:** Work‐related characteristics of the participants (*n* = 304).

Variable	Mean (SD)	Median (Q1, Q3)	Range
Age (years)	31.13 (6.42)	31 (26, 34)	22–54
Average weekly work hours	47.89 (12.84)	48 (38, 54)	15–85
Total clinical experience (years)	6.10 (5.41)	5 (2, 8)	1–30
Emergency dept. experience (years)	3.13 (2.41)	2 (1, 4)	1–16

### 3.2. Job‐Related Stress Scores

Table 3 summarizes the subscale scores of the NSS. The total mean stress score was 62.34 ± 16.45. The highest stress levels were reported in the dimension of “death and dying” (12.73 ± 4.16), followed by “workload” (11.41 ± 3.32). The lowest stress was reported for “lack of support” (4.49 ± 2.15).

**TABLE 3:** Job‐related stress scores and subscales.

Dimension	Mean ± SD	Range
Death and dying	12.73 ± 4.16	2–21
Workload	11.41 ± 3.32	1–18
Treatment uncertainty	9.08 ± 2.97	2–15
Conflict with doctors	9.64 ± 2.92	2–15
Peer conflict	10.09 ± 3.30	1–15
Inadequate preparation	4.89 ± 2.04	0–9
Lack of support	4.49 ± 2.15	0–9

### 3.3. PSC Scores

As shown in Table 4, the average total score on the HSOPSC was 124.39 ± 13.41. The highest mean score was observed in “handoffs and transitions” (14.95 ± 3.15), while the lowest was in “no punitive response to error” (7.56 ± 2.79).

**TABLE 4:** Patient safety culture scores across subdimensions (*n* = 304).

Dimension	Mean ± SD	Range
Frequency of event reporting	8.69 ± 2.12	5–15
Overall perceptions of safety	12.11 ± 2.33	5–18
Supervisor expectations	12.26 ± 2.67	4–20
Organizational learning	9.64 ± 2.30	3–15
Teamwork across units	12.28 ± 3.29	4–19
Communication openness	8.95 ± 1.94	3–13
Feedback about error	9.55 ± 2.64	3–15
Nonpunitive response to error	7.56 ± 2.79	3–15
Staffing	10.83 ± 3.68	4–17
Management support	8.81 ± 2.29	3–14
Teamwork within units	8.76 ± 2.19	3–14
Handoffs and transitions	14.95 ± 3.15	7–25

### 3.4. Correlation Analysis

Due to nonnormal data distribution (Kolmogorov–Smirnov test, *p* < 0.05 for all variables), Spearman correlation coefficients were calculated. There was no significant correlation between job‐related stress (NSS) and total PSC score (*r* = −0.151 and *β* = −0.339, *p* > 0.05). Additionally, no significant association was found between job‐related stress and PSOs (*r* = −0.043, *p* > 0.05). In contrast, a strong and statistically significant positive correlation was observed between PSC and PSOs (*r* = 0.807, *p* < 0.001).

The Spearman correlation coefficients among job‐related stress, PSC, and PSOs are summarized in Table 5.

**TABLE 5:** Spearman correlation coefficients between job‐related stress (NSS), patient safety culture (PSC), and patient safety outcomes (PSO) (*n* = 304).

Variables	Correlation (r)	*p* value	Interpretation
Job‐related stress vs. PSC	−0.151	0.125	Not significant
Job‐related stress vs. PSO	−0.043	> 0.05	Not significant
PSC vs. PSO	0.807	< 0.001	Strong, significant

### 3.5. Mediation Analysis Using PLS‐SEM

**FIGURE 1:**
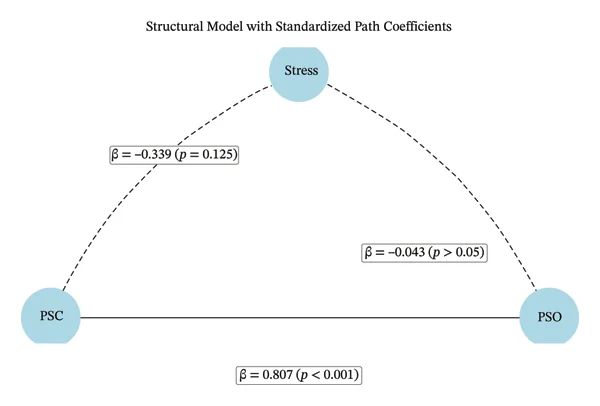
Structural model with standardized path coefficients.

PLS‐SEM was used to assess the mediation hypothesis. The total effect of job‐related stress on PSOs was not significant (total effect = −0.317), and PSC did not mediate the relationship between stress and safety outcomes. The path coefficient from stress to PSC was not significant (*β* = −0.339, *p* > 0.05), and the indirect effect through PSC was minimal. However, PSC had a significant direct effect on PSOs (*β* = 0.807, *p* < 0.001), confirming its important role in shaping safety outcomes. (Figure 1)

## 4. Summary of Findings

• No significant direct or indirect relationship was found between job‐related stress and PSOs.
• PSC was strongly associated with better perceived safety outcomes.
• PSC did not function as a statistical mediator in the stress–outcome relationship in this sample.

## 5. Discussion

This study explored the relationships between job‐related stress, PSC, and PSOs among emergency nurses in two major teaching hospitals in Mashhad, Iran. Contrary to initial expectations, job‐related stress was not significantly associated with PSC or PSO, and the mediation hypothesis was not supported. However, PSC showed a strong and positive association with PSO. This association should be interpreted with caution, as both constructs were derived from the same instrument and may partially reflect shared measurement variance and common method bias rather than a purely theoretical relationship.

Our findings align with international studies that emphasize the importance of a positive PSC for improving safety outcomes, independent of individual stress levels [13, 20, 21]. For instance, studies in Taiwan [13] and Jordan [21] have shown that supportive safety cultures foster error reporting, team collaboration, and communication, leading to fewer adverse events.

The absence of a significant link between job‐related stress and PSO contrasts with previous studies reporting such associations [3, 22]. Several contextual explanations may account for this discrepancy. It is possible that emergency nurses in our setting have developed adaptive coping mechanisms or that informal team support systems buffer the effects of stress on patient care. Furthermore, cultural or organizational norms—such as hierarchical structures or fear of punitive responses—may influence both perceived stress and willingness to report safety issues [10, 17]. These conditions may suppress observable relationships between stress and safety perceptions in middle‐income health systems such as Iran’s.

Although PSC did not function as a mediator in our model, its strong direct effect on PSO suggests that investing in safety culture—through open communication, leadership support, and nonpunitive policies—can yield tangible improvements in patient safety, even if individual stress is not fully addressed. These findings are particularly relevant in resource‐constrained settings where improving organizational culture may be more feasible than large‐scale individual interventions.

Importantly, the null findings in this study contribute to a more balanced and nuanced understanding of the dynamics between stress and safety outcomes. In scientific literature, null or nonsignificant results are often underreported, yet they provide critical insights into where theoretical assumptions may not hold under certain conditions. Our results suggest that the assumed mediation pathway may not be universal and highlight the need for context‐sensitive interventions.

While job‐related stress did not show a statistically significant relationship with PSOs (*β* = −0.043, *p* > 0.05), PSC exhibited a strong and significant positive effect (*β* = 0.807, *p* < 0.001). This suggests that PSC may serve as a more influential and independent predictor of safety outcomes than individual stress levels in emergency settings.

Despite its limitations—including the use of perception‐based PSO measures and a cross‐sectional design—this study provides valuable insights into the interplay of organizational culture and nursing stress in high‐pressure emergency environments. It calls for further research using objective safety indicators, longitudinal designs, and exploration of moderators such as leadership style, resilience, and spiritual coping.

It is important to consider that data were collected through self‐administered questionnaires within the workplace setting. This may have influenced participants’ responses due to social desirability bias or organizational norms related to safety reporting.

### 5.1. Limitations

This study has several limitations that should be acknowledged. A key limitation of this study is the use of subscales from the same instrument (HSOPSC) to measure both PSC and perceived safety outcomes. This may introduce common method bias and inflate observed associations.

First, the cross‐sectional design limits the ability to infer causal relationships between variables. Longitudinal research is needed to determine whether changes in job‐related stress or safety culture precede changes in safety outcomes over time.

Second, the study relied on self‐reported measures for both PSC and PSOs. While validated instruments were used, subjective perceptions may not fully capture actual safety incidents. Future research should incorporate objective outcome metrics such as incident reports or clinical error tracking.

Third, although PLS‐SEM was suitable given the sample size and nonnormal data distribution, it lacks global fit indices and is less robust than covariance‐based SEM for testing mediation models. This methodological choice is acknowledged, and future studies may benefit from using confirmatory approaches to validate these findings.

Fourth, several potential confounding factors were not measured—such as workload, nurse‐to‐patient ratios, and institutional reporting policies—all of which may influence both stress and safety culture. Including such variables in future models would help clarify their potential moderating or mediating roles.

Lastly, the study was conducted in two public teaching hospitals in a single city in Iran, which may limit the generalizability of findings to other healthcare settings or cultural contexts.

### 5.2. Implications for Practice and Research

From a clinical perspective, the findings suggest that strengthening PSC may have a more direct impact on improving safety outcomes than focusing solely on individual stress reduction. Practical strategies include fostering open communication, implementing nonpunitive error reporting systems, and enhancing leadership support in EDs.

The findings underscore the critical role of PSC in improving perceived safety outcomes, regardless of job stress levels. Healthcare administrators should prioritize interventions aimed at cultivating open communication, team cohesion, and nonpunitive approaches to error reporting. Future research should incorporate objective safety metrics, expand to multisite samples, and explore moderating variables such as resilience, leadership style, and spiritual coping—especially in culturally rich and religious settings like Iran.

Rather than confirming a universal causal pathway, the findings suggest that the relationship between job‐related stress and PSOs may be context dependent, particularly in resource‐constrained healthcare systems.

## 6. Conclusion

This study examined the interrelationships between job‐related stress, PSC, and PSOs in emergency nursing settings. Contrary to our initial hypothesis, job‐related stress did not have a statistically significant effect on either PSC or PSO, and no mediating role of PSC was observed. These null findings offer an important counterbalance to existing literature and suggest that the impact of stress on safety outcomes may not be uniform across all healthcare contexts.

However, the strong and significant association between PSC and PSO reinforces the pivotal role of organizational culture in shaping safety outcomes—even in high‐pressure and resource‐constrained environments. These findings suggest that healthcare administrators may achieve greater safety improvements by investing in safety culture interventions, such as enhancing communication openness, reducing punitive responses to errors, and strengthening leadership support.

Reporting nonsignificant results contributes to a more complete and realistic understanding of complex healthcare dynamics. Future research should explore potential moderating variables—such as resilience, leadership style, or staffing policies—and should prioritize longitudinal designs and objective safety metrics to better capture causal relationships.

## Author Contributions

Alam Abbasi Yazdi and Javad Moghri contributed to the conceptualization and design of the study. Jamshid Jamali supervised the clinical aspects and provided methodological guidance. Elahe Hooshmand conducted the data collection, performed the statistical analysis, and drafted the initial manuscript. All authors critically reviewed and revised the manuscript for important intellectual content.

## Funding

This research did not receive any specific funding.

## Disclosure

All authors have approved the final version for submission.

## Ethics Statement

This research was approved by Ethics Committee of Mashhad University of Medical Sciences (IR.MUMS.REC.1401.409).

## Conflicts of Interest

The authors declare no conflicts of interest.

## Data Availability

The data that support the findings of this study are available from the corresponding author upon reasonable request, subject to ethical and confidentiality considerations.

## References

[1] Mistri I. U. , Badge A. , and Shahu S. , Enhancing Patient Safety Culture in Hospitals, Cureus. (2023) 15, no. 12, 10.7759/cureus.51159. PMC10811440 38283419

[2] Donaldson L. , Ricciardi W. , Sheridan S. , and Tartaglia R. , Textbook of Patient Safety and Clinical Risk Management, 2021. 36315660

[3] Garcia C. L. , Abreu L. C. , Ramos J. L. S. et al., Influence of Burnout on Patient Safety: Systematic Review and Meta-Analysis, Medicina. (2019) 55, no. 9, 10.3390/medicina55090553. PMC6780563 31480365

[4] Rodziewicz T. L. , Houseman B. , and Hipskind J. E. , Medical Error Reduction and Prevention, 2018. 29763131

[5] Gmayinaam V. U. , Nortey A. N. , Sedode S. et al., Work-Related Stress Among Nurses: A Comparative Cross-Sectional Study of Two Government Hospitals in Ghana, BMC Public Health. (2024) 24, no. 1, 10.1186/s12889-024-19757-3. PMC11334492 39164666

[6] Mossburg S. E. and Dennison Himmelfarb C. , The Association Between Professional Burnout and Engagement With Patient Safety Culture and Outcomes: A Systematic Review, Journal of Patient Safety. (2021) 17, no. 8, e1307–e1319, 10.1097/pts.0000000000000519. 29944601

[7] Batalha E. M. SdS. , Borges E. MdN. , and Melleiro M. M. , Association Between Patient Safety Culture and Professional Quality of Life Among Nursing Professionals, Revista da Escola de Enfermagem da USP. (2024) 58, 10.1590/1980-220x-reeusp-2023-0359en. PMC11268136 38985821

[8] de Lima Garcia C. , Bezerra I. M. P. , Ramos J. L. S. , do Valle J. E. T. M. R. , Bezerra de Oliveira M. L. , and Abreu L. C. , Association Between Culture of Patient Safety and Burnout in Pediatric Hospitals, PLoS One. (2019) 14, no. 6, 10.1371/journal.pone.0218756. PMC6590886 31233543

[9] Organization WH , Global Patient Safety Report 2024, 2024, World Health Organization.

[10] Zabin L. M. , Qaddumi J. , Ghawadra S. F. , and Battat M. M. , Job Stress and Patient Safety Culture: A Qualitative Study Among Hospital Nurses in Palestine, BMC Nursing. (2025) 24, no. 1, 10.1186/s12912-025-02993-2. PMC11934625 40128807

[11] Rattray J. , Miller J. , Pollard B. et al., A Model of Occupational Stress to Assess Impact of COVID-19 on Critical Care and Redeployed Nurses: A Mixed-Methods Study, Health and Social Care Delivery Research. (2024) 13, 1–32, 10.3310/pwrt8714. 39708055

[12] Zabin L. M. , Zaitoun R. S. , Sweity E. M. , and de Tantillo L. , The Relationship Between Job Stress and Patient Safety Culture Among Nurses: A Systematic Review, BMC Nursing. (2023) 22, no. 1, 10.1186/s12912-023-01198-9. PMC9926568 36782195

[13] Chen I. C. and Li H. H. , Measuring Patient Safety Culture in Taiwan Using the Hospital Survey on Patient Safety Culture (HSOPSC), BMC Health Services Research. (2010) 10, no. 1, 10.1186/1472-6963-10-152. PMC2903582 20529246

[14] Nie Y. , Mao X. , Cui H. , He S. , Li J. , and Zhang M. , Hospital Survey on Patient Safety Culture in China, BMC Health Services Research. (2013) 13, no. 1, 10.1186/1472-6963-13-228. PMC3701538 23800307

[15] Bakar R. M. , Khaerah Y. , Hidayati N. , and Hamid A. N. , The Role of Organizational Culture in Moderating Effect of Emotional Labor Strategies on Nursing Professionalism, Nurse Media Journal of Nursing.(2022) 12, no. 1, 122–132, 10.14710/nmjn.v12i1.35626.

[16] Wu H. , Qi K. , Luan B. , Liu Z. , and Zhao Q. , Association Between Occupational Stress and Mental Health of Nurses During the COVID-19 Pandemic: A Cross-Sectional Research, Nursing Open. (2023) 10, no. 12, 7694–7702, 10.1002/nop2.2010. 37767901 PMC10643833

[17] Zabin L. M. , Qaddumi J. , and Ghawadra S. F. , The Relationship Between Job Stress and the Perception of Patient Safety Culture Among Palestinian Hospital Nurses, BMC Nursing. (2025) 24, no. 1, 10.1186/s12912-025-03009-9. PMC11963279 40170149

[18] Sani M. M. , Jafaru Y. , Ashipala D. O. , and Sahabi A. K. , Influence of Work-Related Stress on Patient Safety Culture Among Nurses in a Tertiary Hospital: A Cross-Sectional Study, BMC Nursing. (2024) 23, no. 1, 10.1186/s12912-023-01695-x. PMC10829317 38291420

[19] Hassmiller S. B. and Wakefield M. K. , The Future of Nursing 2020–2030: Charting a Path to Achieve Health Equity, Nursing Outlook. (2022) 70, no. 6, S1–S9, 10.1016/j.outlook.2022.05.013. 36446536

[20] Kim J. , An K. , Kim M. K. , and Yoon S. H. , Nurses’ Perception of Error Reporting and Patient Safety Culture in Korea, Western Journal of Nursing Research. (2007) 29, no. 7, 827–844, 10.1177/0193945906297370. 17636243

[21] Alabed H. R. WeM. , Work Environment and Job Satisfaction Among Nurses in Jordan: A Systematic Literature Review, British Journal of Healthcare Management. (2023) 29, no. 4, 1–7, 10.12968/bjhc.2021.0128.

[22] Roberts R. K. and Grubb P. L. , The Consequences of Nursing Stress and Need for Integrated Solutions, Rehabilitation Nursing. (2014) 39, no. 2, 62–69, 10.1002/rnj.97. 23696492 PMC4664060

